# So Many Brands and Varieties to Choose from: Does This Compromise the Control of Food Intake in Humans?

**DOI:** 10.1371/journal.pone.0125869

**Published:** 2015-04-29

**Authors:** Charlotte A. Hardman, Danielle Ferriday, Lesley Kyle, Peter J. Rogers, Jeffrey M. Brunstrom

**Affiliations:** 1 Department of Psychological Sciences, University of Liverpool, Liverpool, United Kingdom; 2 Nutrition and Behaviour Unit, School of Experimental Psychology, University of Bristol, Bristol, United Kingdom; Swansea University, UNITED KINGDOM

## Abstract

The recent rise in obesity is widely attributed to changes in the dietary environment (*e*.*g*., increased availability of energy-dense foods and larger portion sizes). However, a critical feature of our “obesogenic environment” may have been overlooked - the dramatic increase in “dietary variability” (the tendency for specific mass-produced foods to be available in numerous varieties that differ in energy content). In this study we tested the hypothesis that dietary variability compromises the control of food intake in humans. Specifically, we examined the effects of dietary variability in pepperoni pizza on two key outcome variables; i) compensation for calories in pepperoni pizza and ii) expectations about the satiating properties of pepperoni pizza (expected satiation). We reasoned that dietary variability might generate uncertainty about the postingestive effects of a food. An internet-based questionnaire was completed by 199 adults. This revealed substantial variation in exposure to different varieties of pepperoni pizza. In a follow-up study (*n*= 66; 65% female), high pizza variability was associated with i) poorer compensation for calories in pepperoni pizza and ii) lower expected satiation for pepperoni pizza. Furthermore, the effect of uncertainty on caloric compensation was moderated by individual differences in decision making (loss aversion). For the first time, these findings highlight a process by which dietary variability may compromise food-intake control in humans. This is important because it exposes a new feature of Western diets (processed foods in particular) that might contribute to overeating and obesity.

## Introduction

Given the substantial health and economic costs of obesity [1,2], there has been extensive effort to identify specific causal associations [3,4,5]. Associated with the increasing prevalence of overweight and obesity there have been changes in our dietary environment. In particular, researchers have considered the role of larger food portion sizes and the increased availability of energy-dense foods [6,7]. However, other changes have also taken place. In the United States, the number of new food products rose substantially between 1970 and 1995, and sweeteners and fat substitutes have become commonplace [8]. Therefore, for any one type of food, there are often many varieties that can differ considerably in their energy content. There are several reasons to suspect that this increasing “dietary variability” might compromise our control of food intake. However, to date, this prospect has remained unexplored.

It is well established that much of our dietary behaviour is based on prior experience [9,10]. In particular, the orosensory properties of a food may serve as a cue (the conditioned stimulus; CS) that enables us to predict the postingestive consequences of its consumption (the unconditioned stimulus; US). In non-human animals the predictive association between orosensory cues (*e*.*g*., sweet tastes, viscosity, fat) and caloric postingestive consequences can be manipulated systematically [11,12,13,14,15,16,17]. Sweetness is a basic taste that has the capacity to generate a primitive appetitive response in newborns [18]. However, the preferred level of sweetness varies considerably, implying that even this initial innate liking can be modified over time [19]. Indeed, rats that are repeatedly exposed to low-energy sweeteners may lose the capacity to use sweetness as a cue to predict caloric intake. Evidence across several studies has been interpreted as showing that such “non-predictive” flavour-nutrient associations increase food intake, bodyweight, and adiposity [11,12,13,14,15,16,17]. Furthermore, after exposure to low-energy sweeteners, non-human animals show reduced sensitivity to calories in a novel and sweet food. Specifically, at a subsequent “test meal”, and relative to control animals, they show less adjustment for the calories in this novel food (*i*.*e*., poor “caloric compensation”) [14]. Recently, the same expression of aberrant dietary control has been observed in animals that have been exposed to fat replacers [20]. The extent to which these manipulations mirror the effects of natural dietary variability in humans remains unclear, however. Nonetheless, dietary variability may degrade our ability to use the visual and orosensory properties of a specific food to anticipate its energy content. In the present study, we tested this idea by examining evidence for reduced sensitivity to the calories in a food that has high dietary variability.

There is increasing consensus that the control of meal size in humans is learned and expressed in cognitive activity associated with meal planning, before a meal begins [21]. Most (~ 86%) meals are self-selected and then consumed in their entirety [22,23,24]. Consistent with a role for pre-meal planning, we have shown previously that people have very clear expectations and beliefs about the satiation that foods are likely to confer [25,26]. This “expected satiation” is an excellent predictor of portion-size selection and food intake [24,26,27]. Importantly, these expectations are dynamic and change over time. For example, studies have shown that expected satiation increases as a food becomes more familiar [28,29]. In relation to learning, dietary variability could be highly relevant because it might challenge our capacity to form a separate expectation about the satiating consequences of each individual brand and variety of a specific mass-produced food or meal [10]. Accordingly, in the present study we explored the effects of dietary variability on a measure of expected satiation. In keeping with the findings from the non-human animal studies, we predicted that high levels of dietary variability within a specific food would be associated with lower expected satiation for that food. In addition to influencing expected satiation, dietary variability might also generate uncertainty about these expectations. We sought to quantify this uncertainty using a measure of “expected-satiation confidence”, which is based on methods used previously in our laboratory [28].

A second objective was to explore the extent to which individual differences in decision making moderate responses to dietary uncertainty. An important and general characteristic of human decision making is that people select options that appear to be irrational. In particular, Prospect Theory [30,31] predicts that people value potential losses as more important than corresponding gains. This “loss aversion” is relevant here because it is exposed in decisions that involve uncertainty [32]. For example, people may avoid a gamble in which they are equally likely to either lose $10 or win $15, even though the “expected utility” (the net gain over many trials) of the gamble is positive ($2.50). Despite the importance of loss aversion it has tended to be studied in a rather limited domain—typically in the context of monetary gambling. This focus on monetary decision making reflects the convenient use of money as a common valued commodity across participants. However, loss aversion has also been found in other species [33], suggesting a more general strategy that confers a benefit, perhaps in relation to foraging and the procurement of food.

The role of loss aversion as a potential moderator of dietary choices and food intake remains unexplored. We also note that people differ markedly in their monetary loss aversion [34,35,36]. Therefore, one possibility is that this individual difference generalises to other forms of decision making around objects that also have value. Our interest in loss aversion is justified because we can envisage two ways in which it might influence food intake. First, people may be loss averse with respect to the prospect of overconsumption at a test meal. It is widely acknowledged that meal size is governed by an anticipation of the aversive effects of overconsumption (we rarely eat to physical capacity) [37,38]. One possibility is that loss averse people are especially motivated to avoid the negative consequences of overconsumption (they value the status quo). If dietary variability generates uncertainty about these negative effects then this may promote conservative dietary decisions leading to a reduction in test-meal intake. Second, people may be loss averse with respect to concerns about feeling hungry between meals. If dietary variability promotes uncertainty about the satiating capacity of a food then this may motivate people to consume a larger meal in a subsequent assessment of dietary compensation. They do so because they are motivated to avoid the negative effects of under-consumption (*i*.*e*., feeling hungry).

In summary, the overarching aim of this exploratory study was to quantify the effects of dietary variability in a commonly consumed food. Pepperoni pizza was selected because it is extremely common and widely available throughout the UK. We sought to test three hypotheses. Firstly, we predicted that exposure to different types of pepperoni pizza (a wide range of energy contents) would be associated with reduced compensation for calories in this food at a subsequent test meal (here we used an established compensation index, known as a COMPX score [39,40]). Secondly, we predicted that high levels of dietary variability would be associated with lower expected satiation for pepperoni pizza. Finally, we predicted that the effects of low expected-satiation confidence (*i*.*e*., uncertainty) on COMPX scores and expected satiation would be moderated by individual differences in loss aversion.

## Methods

Our study comprised two distinct stages. In Stage 1, a questionnaire was administered to quantify individual differences in dietary variability for pepperoni pizza. In Stage 2, participants attended laboratory test sessions to assess the effects of pizza variability on our key outcome measures, COMPX and expected satiation.

### Stage 1: Measurement of pizza variability

#### Respondents

In total, 199 individuals [70 male and 129 female; mean age (SD) = 25.1 (6.7) years] completed an internet-based questionnaire. Respondents were recruited from the population of Bristol, UK, via an online database of volunteers belonging to the research group. These volunteers had previously joined the database following advertisements posted within the university and the wider community (e.g., in local newspapers) for participants for eating behaviour research studies. At the time of conducting this study, 5512 volunteers (57% female) were registered on the database. Volunteers comprised university students, staff and members of the public, and a wide range of ages was represented (18–72 years).

Vegetarians, vegans, current dieters, and smokers were excluded from the study, together with individuals declaring food allergies or intolerances. We also excluded anyone who had not eaten pizza in the last year. Respondents were entered into a prize draw to win £100 (Pounds Sterling) worth of vouchers. As a cover story, they were informed that the objective of the study was to explore the effects of mood on appetite for food. Ethical approval was granted by the University of Bristol’s Faculty of Science Human Research Ethics Committee. Informed consent was obtained from all respondents prior to completing the questionnaire.

#### Measures

In the first instance we compiled a list of the types of pepperoni pizza that are available to consumers in the local region. Our research incorporated information based on products sold in the UK and distributed by the seven largest supermarkets and by nationally established pizza takeaway and restaurant chains in the local Bristol area. Data were collected by consulting the websites of the supermarkets and restaurants between May and June 2012. The energy content of every regular-sized pepperoni pizza (8- to 12-inch diameter) was recorded. Pizzas that were intended for more than one person and miniature pizzas were not included. This yielded a total of 71 different types of pepperoni pizza across 14 brands (Table 1). In cases where there was more than one type of pepperoni pizza per brand (e.g., there were ten different Tesco own/store-branded pepperoni pizzas), we computed the average pizza energy content (kcal) for this brand. As shown in Table 1, these values ranged from 501 to 1909 kcal per pizza brand, confirming that substantial dietary variability exists across brands and varieties (381%).

**Table 1:** Mean energy content (*SD* in parentheses) of the 14 brands of pepperoni pizza.

Pizza brand	Number of pepperoni pizzas per brand	Mean (*SD*) pizza energy content in kcal
1. Weight Watchers	1	501 (-)
2. Pizza Express	10	721 (201)
3. Dr Oetker	1	873 (-)
4. Asda (own/store brand)	7	947 (242)
5. Waitrose (own/store brand)	6	983 (343)
6. Co-operative (own/store brand)	2	989 (249)
7. Marks and Spencers (own/store brand)	1	1071 (-)
8. Sainsburys (own/store brand)	8	1107 (223)
9. Goodfellas	4	1107 (98)
10. Tesco (own/store brand)	10	1109 (188)
11. Morrisons (own/store brand)	6	1124 (221)
12. Pizza Hut	5	1350 (289)
13. Chicago Town	2	1563 (499)
14. Dominos	8	1909 (560)
**All brands**	**71**	**1133 (433)**

Note. Only regular-sized pizzas (8–12 inch diameter) were included. *SD* not computed where only one pizza per brand. Own/store brand pepperoni pizzas are manufactured and sold by the aforementioned supermarket.

In the first part of the online questionnaire, respondents provided information about their gender, age, height, weight, and their frequency of consumption of pizza in the last year. They then reported the size of their typical portion of a medium 10-inch pizza (response options were one quarter, one half, three-quarters or one whole pizza). To assess prior experience of eating different brands of pepperoni pizza, respondents were then shown images of each of the 14 brands of pepperoni pizza (Table 1). In turn, they were asked, “Is it likely that you have eaten a (insert brand name) pepperoni pizza within the last year?” (response options were “yes” or “no”).

For each respondent, the number of pepperoni pizza brands consumed over the past year was computed by counting the number of “yes” responses (minimum = 1, maximum = 14). With this information we then computed the mean energy content of pepperoni pizzas consumed by each respondent, henceforth referred to as “pizza energy content”. In order to quantify pizza variability, we made an *a priori* decision to use each respondent’s inter-quartile range (IQR; the difference between the 25^th^ and 75^th^ percentiles) of the energy content of the pizza brands consumed—here referred to as a measure of “pizza variability”.

In the next part of the online questionnaire, respondents completed a monetary gambling task [35] to measure individual differences in loss aversion. Participants were asked to imagine that they had been given £50 (Pounds Sterling) in cash. They were then presented with 49 gambles and in each gamble they were asked to bet on the outcome of a hypothetical coin toss. Respondents were told that if the coin turned up “heads” then they would lose some money (*e*.*g*., “Heads you lose £10”) and if it turned up “tails” then they would win some money (*e*.*g*., “Tails you win £45”). Respondents were required to accept or reject each hypothetical gamble by selecting the appropriate response on the screen. The amounts of money that could be lost or won varied across each gamble and were randomly sampled from a matrix which consisted of seven potential gains ranging from £20 to £50, and seven potential losses ranging from -£0 to -£30. Gains and losses went up in increments of £5.

Expected values (EV) for each gamble were computed as follows; EV = 0.5(gain) + 0.5(loss) [35]. For example, the EV for the gamble “Heads you lose £10, Tails you win £45” would be 17.5. Across the 49 gambles, EVs ranged from a minimum of -5.0 to a maximum of +25.0. For each respondent, EVs were plotted against corresponding binary responses (*i*.*e*., accept or reject). Probit analysis was used to fit a sigmoid function to determine the EV at which the respondent was equally likely to accept or reject the gamble (the indifference point). A positive indifference point indicates that the respondent tended to gamble only when potential losses were smaller than potential gains. A large and positive indifference point indicates a high degree of loss aversion.

In the final part of the questionnaire, respondents completed the dietary restraint and disinhibition scales of the Three Factor Eating Questionnaire (TFEQ; [41]), and the Depression, Anxiety and Stress Scale (DASS; [42]). The latter was included for the purpose of the cover story and the data were not analysed.

#### Stage 1 results

**Fig 1:**
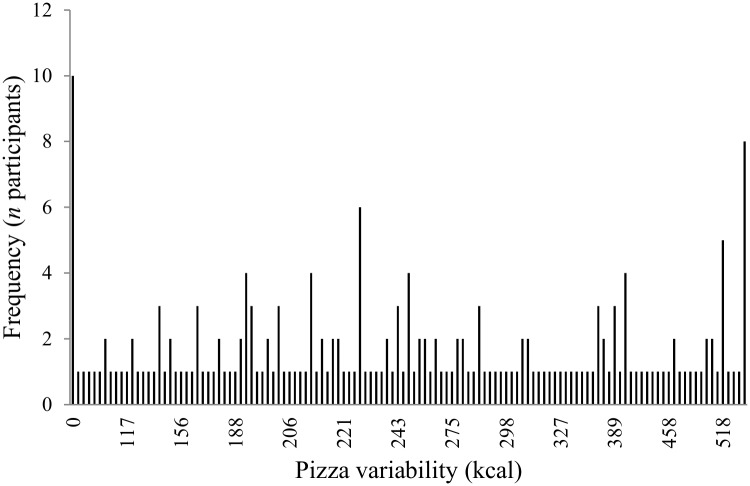
Individual differences in pizza variability for respondents (*N* = 199) in Stage 1. *Note*. Individual scores for pizza variability were computed using the IQR of the energy content of the pizza brands consumed by each respondent over the past year.

On average, respondents reported having eaten 5.2 (SD = 2.4) different brands of pepperoni pizza over the past year. The mean energy content of these pizzas was 1208 (SD = 170) kcal. The mean variability in pizza energy content was 271 (SD = 143) kcal. Individual values for variability ranged from 0 (i.e., no variability; the respondent had consumed only one brand of pepperoni pizza) through to 594 kcal. These values are displayed graphically in Fig 1. A Kolmogorov-Smirnov test confirmed that the data were not normally distributed (KS = 0.12, p <.001). Non-parametric correlation coefficients indicated that TFEQ-dietary restraint was positively correlated with pizza variability (Spearman’s rho = .18, p = .012). However, neither age, BMI, TFEQ-disinhibition nor loss aversion correlated significantly with pizza variability (all p values >.08).

In summary, the results of Stage 1 confirmed that (i) substantial dietary variability exists across brands and varieties of a single food type, pepperoni pizza (range in energy contents from 501 to 1909 kcal, 381%), and (ii) individuals differ considerably in the extent to which they are exposed to this variability (pizza variability ranged from 0 to 594 kcal).

### Stage 2: Laboratory observations

#### Participants

Power analysis (G*Power version 3.1.3) indicated that we would need to recruit 61 participants in order to detect medium-sized correlations (r = .35) between the key variables at an alpha level of 0.05 (two-tailed) with 80% power. The magnitude of the predicted effect was based on our previous work on the association between expected satiation and food familiarity (r = 0.37) [29]. To allow for drop outs, we aimed to slightly over-recruit by selecting 66 participants.

Participants for the laboratory observations were selected from the pool of 199 respondents who successfully completed the online questionnaire. Within this smaller sample (*n* = 66), we ensured that the variability on three key variables (pizza variability, pizza energy content, and number of pizza brands eaten) was maximized. This was achieved using an iterative algorithm. Firstly, responses associated with each of the three variables were standardized and converted to absolute values. For each individual, these were then summed to produce a composite total residual. The individual with the smallest total residual was removed and this iterative process was repeated until the required number of participant remained.

In total, 23 men and 43 women participated in the laboratory observations. All participants gave written informed consent to participate in the laboratory observations and received £15 (Pounds Sterling) as remuneration for their assistance. The cover story, that the study was researching the effects of mood on appetite for food, was maintained.

#### Test foods

In a typical study designed to measure caloric compensation, a fixed amount of a food is given as a “preload” and subsequent ad libitum intake of other foods (the “test meal”) is measured [43]. The preload food was a standard pepperoni 10-inch pizza (“Italian Pepperoni” supplied by Sainsbury’s Supermarkets Limited; energy density = 294 kcal per 100 g). This brand of pizza was specifically chosen because its total energy content was close to the median based on the 14 brands of pepperoni pizza that were identified in Stage 1 (Table 1). The preload was standardised as one quarter of a commercially available pizza [mean (SD) energy content = 256 (33) kcal]. It was served on a white plate with a 255-mm diameter.

The test meal comprised tortilla chips (“Basics Tortilla Chips”, Sainsbury’s Supermarkets Limited; 489 kcal per 100 g) and dark chocolate chip cookies (“Basics Cookies”, Sainsbury’s Supermarkets Limited; 496 kcal per 100 g). Participants received 200 g of each food in separate 1-litre Pyrex bowls (1970 kcal in total). Consistent with previous research [44], the cookies were broken into pieces to limit the opportunity to self-monitor intake by counting whole cookies.

#### Measures

Ratings of hunger, fullness, expected and actual (pre/post-tasting) liking for the preload, and liking for test meal, were obtained using computerised 100-mm visual analogue scales (VAS). All scales used the same end anchor points—“not at all” and “extremely”. The rating of expected-satiation confidence was obtained using a 100-mm VAS that was headed “Are you confident about the extent to which the pizza would fill you up?” The anchor points were “not at all” and “extremely”.

Our measure of expected satiation was based on a “method of constant stimuli”, described in detail elsewhere [25]. The associated computer code was written and executed using MATLAB (version R2009a). A physical portion of the pizza preload (the standard food) was placed in front of the participant. An image of a different “comparison food” was displayed on a computer screen. Spaghetti Bolognese was chosen as the comparison food because we have shown that it is highly familiar to a sample drawn from a similar population [25]. Over 56 trials, the participants pressed the left or right arrow key on the computer keyboard to indicate which of the two foods (*i*.*e*., the pizza preload or the spaghetti Bolognese) would leave them feeling fuller immediately after consumption. In each trial, an algorithm selected the portion of spaghetti Bolognese from a series of 50 images depicting portions ranging from 20 kcal to 1000 kcal in linear 20-kcal steps. These images had previously been taken using a high-resolution digital camera mounted above a 255-mm diameter white plate (particular care was taken to ensure lighting and angle consistency across the images). Across trials, the algorithm selected specific portion sizes of spaghetti Bolognese based on previous responses made by the participant. Portion sizes were chosen to maximise the accuracy of a “point of subjective equality” (PSE) that can be estimated at the end of the block of trials. For each participant, the PSE represents the portion size around which the standard (pizza) and the comparison (spaghetti Bolognese) are equally likely to be selected. As such, it provides an estimate of the amount of spaghetti Bolognese (kcal) that is expected to be equally filling as the fixed pizza preload. Higher values indicate that the pizza has greater expected satiation.

Consistent with the cover study, the Positive and Negative Affect Schedule (PANAS) was employed to assess mood [45]. The scale comprises 10 positive items (*e*.*g*., interested, excited, determined) and 10 negative items (*e*.*g*., irritable, distressed, afraid). Participants rated each item on a five point scale (1 = “very slightly” or “not at all” and 5 = “extremely”) on the basis of how they felt at the present moment. This measure and all other ratings were implemented on a PC using custom software written in Visual Basic (Microsoft version 6.0).

#### Procedure

Participants attended two 60-minute lunchtime sessions, spaced exactly one week apart. In one session they consumed the pizza preload followed by the ad libitum test meal. In the other session they received only the ad libitum test meal. The order of the sessions was counterbalanced across participants. At the beginning of the first session the participants provided written informed consent. Participants were asked to abstain from eating and drinking (except water) for 3 hours prior to each test session. All confirmed compliance with this instruction.

In the preload session, participants completed baseline appetite ratings followed by the PANAS. The pepperoni pizza preload was presented together with 250 ml of water. Before tasting the pizza they rated its expected liking. After taking a small bite, they then rated their actual liking and then completed the expected-satiation task. Finally, before consuming the pizza in its entirety, they rated their expected-satiation confidence and completed a further set of appetite ratings. After a 20-minute period the participants were presented with the test meal. They were instructed to eat *ad libitum* until they felt comfortably full. Appetite ratings were completed pre- and post-consumption.

The no-preload session was identical to the preload session with the exception that no pizza preload was presented, and participants did not rate liking, expected satiation or expected-satiation confidence. Instead, these tasks were replaced by the opportunity to engage in light reading with timings matched across the two conditions.

At the end of the second test session, participants rated their liking for the test meal. Their height and weight was measured, and they were then debriefed and offered remuneration for their assistance.

#### Analyses

To calculate a compensation index (COMPX), for each participant, the difference in test-meal intake (kcal) between the two test sessions was first computed by subtracting intake in the preload session from intake in the no-preload session. This difference score was then divided by the energy content of the pizza preload and transformed to a percentage [40]. A lower COMPX score indicates lower sensitivity to calories in the preload at the test meal (i.e., less adjustment of intake at the test meal on the preload day relative to the no-preload day).

Descriptive statistics were computed for all outcome variables. A 2x2 repeated measures analysis of variance (ANOVA) was used to examine differences in intake of the two test-meal foods (tortilla chips, cookies) during the two test sessions (preload, no-preload). Differences in total energy intake (*i*.*e*., preload + test meal) was also examined across the two test sessions using a paired *t* test. Composite appetite scores were calculated using the following formula: (hunger + (100-fullness))/2. Repeated measures ANOVA was used to examine changes in appetite scores over time across sessions.

Separate linear-regression analyses were conducted with COMPX score and expected satiation as outcome variables, respectively. For each outcome variable, the primary model included the following predictor variables (i) pizza variability (ii) pizza energy content, and (iii) loss aversion (measures i, ii, and iii, taken from responses elicited during Stage 1). We included pizza energy content (*i*.*e*., the mean energy content of all pepperoni pizzas consumed by each participant over the past year) in order to examine the effects of variability independent of a general tendency to consume pizzas with a higher energy content. In order to address the secondary objective of our study (*i*.*e*., to determine the extent to which individual differences in decision making moderate responses to dietary uncertainty), for each outcome variable in turn, a second regression model included the following additional predictors; (iv) expected-satiation confidence, and (v) a term for the interaction between expected-satiation confidence and loss aversion. The predictor variables, expected-satiation confidence and loss aversion, were mean-centered (*i*.*e*., the mean of each variable was subtracted from the original scores). The interaction term was then computed by multiplying mean-centered expected-satiation confidence with mean-centered loss aversion. Finally, to control for the influence of more general aspects of previous pizza exposure, for each outcome variable in turn, the following variables were entered into a third regression model; (vi) frequency of pizza consumption, (vii) usual pizza portion size, and (viii) the number of pizza brands eaten over the previous year. Analyses were conducted using IBM SPSS version 19.

## Results

Descriptive characteristics of the 66 participants (65% female) selected for laboratory-based observation are shown in Table 2.

**Table 2:** Descriptive characteristics of participants included in the Stage 2 laboratory observations (*n* = 66; 65% female).

	Mean (*SD*)
Age (y)	27.4 (8.5)
BMI (kg/m^2^)	22.7 (3.4)
TFEQ-restraint (0–21)	6.6 (3.9)
TFEQ-disinhibition (0–16)	6.4 (3.4)
Loss aversion (indifference point; -5.0 to +25.0)	9.0 (5.4)
Pizza variability (kcal)	271 (167)
Pizza energy content (kcal)	1218 (223)
Number of pepperoni pizza brands consumed (1–14)	4.9 (3.2)
Usual pizza portion size^a^	0.75 (0.26)
Frequency of pizza consumption^b^	4.3 (1.1)

^a^ Response options were one quarter, one half, three-quarters or one whole of a medium-sized 10-inch pizza.

^b^ 7-point scale; 1 = less than once per year, 2 = once per year, 3 = every 2 to 3 months, 4 = once per month, 5 = fortnightly, 6 = once per week, 7 = every day.

Note. Data on all variables (with the exception of BMI) were carried forward from the responses that these participants’ provided during the Stage 1 questionnaire.

### Energy intake and appetite

Participants consumed significantly more of the test meal on the no-preload day relative to the preload day, *F*(1,65) = 54.5, *p* <.001, and there was also a significant food by session interaction, *F*(1,65) = 4.5, *p* = .04. Post hoc comparisons indicated that intake of tortilla chips and cookies did not differ significantly on the preload day [means (*SD*s) = 162 (126) and 158 (101) kcal, respectively, *t*(65) = 0.2, *p* = .84]. On the no-preload day, there was a trend for greater intake of tortilla chips relative to cookies, however this did not reach statistical significance [256 (198) and 213 (138) kcal, respectively, *t*(65) = 1.5, *p* = .13].

**Fig 2:**
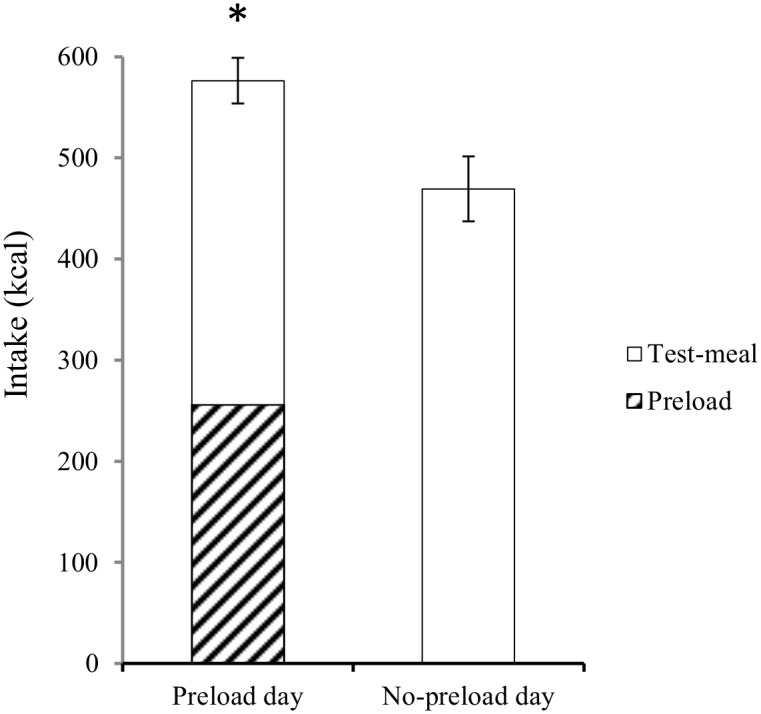
Mean total intake (kcal) on the pizza preload and no-preload test days. Errors bars represent ± 1 *SE* from the mean. * Total intake (preload + test-meal) significantly higher on preload day relative to no-preload day, *p* <.001.

Total intake on the preload day (*i*.*e*., preload + test meal) was significantly greater relative to the no-preload day, *t*(65) = -5.3, *p* <.001 (Fig 2). The mean COMPX score was 58.1% (*SD* = 64.2). We found little evidence that COMPX scores were affected by the order of the two test sessions [Preload session first, mean (*SD*) = 53.0 (69.6)%; No-preload session first, = 63.0 (59.2)%; *p* = .53] and COMPX scores were very similar in males and females [59.0 (75.7)% and 57.7 (58.0)%, respectively; *p* = .94]. COMPX scores and test-meal liking ratings were not significantly correlated (*r* = .21, *p* = .1).

**Fig 3:**
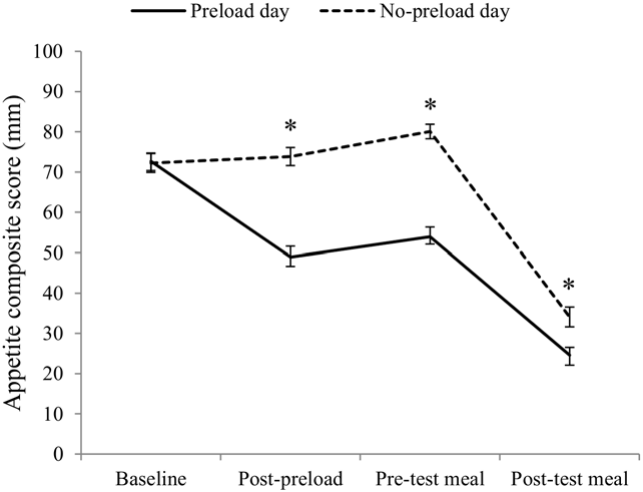
Mean appetite composite scores (100-mm VAS) on the preload and no-preload test days across measurement time points. Errors bars represent ± 1 *SE* from the mean. Appetite composite scores were calculated using the following formula: (hunger + (100-fullness))/2. * significant difference between preload day and no-preload day, *p* <.001.

In both sessions, composite appetite scores decreased significantly from baseline to post-test meal, *F*(3, 195) = 195.4, *p* <.001. There was also a significant session by time interaction, *F*(3, 195) = 37.8, *p* <.001 (Fig 3). *Post-hoc* paired *t* tests indicated no significant difference in appetite score between the test sessions at baseline (*p* = .87). Appetite composite scores were significantly lower in the preload session relative to the no-preload session immediately after consuming the pizza preload (*t* = -7.6, *df* = 65, *p* <.001), at pre-test meal (*t* = -8.9, *df* = 65, *p* <.001) and at post-test meal (*t* = -4.1, *df* = 65, *p* <.001).

### Relationship between variability and COMPX

**Fig 4:**
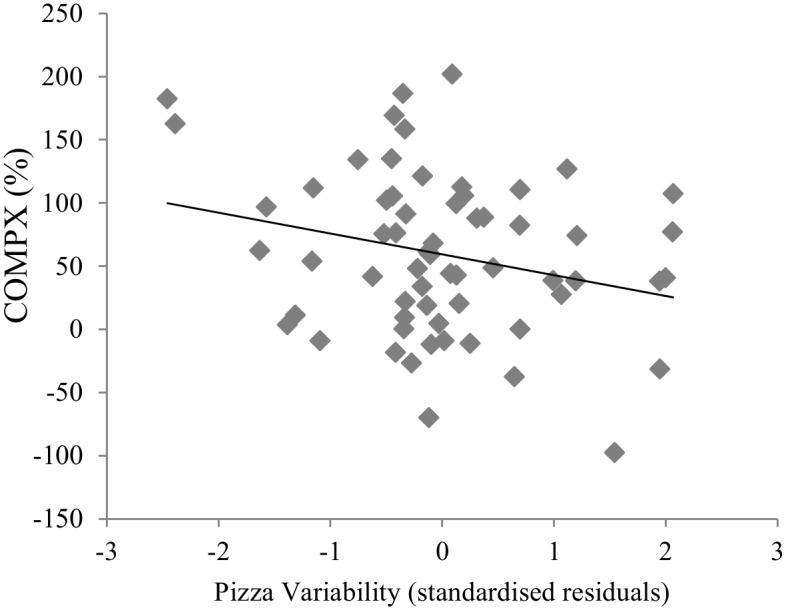
Scatterplot and linear best fit to show the association of pizza variability with COMPX. Values for pizza variability are standardized residuals adjusted for pizza energy content and loss aversion.

The primary regression model accounted for 14% of the variance in COMPX, *F*(3, 61) = 3.2, *p* = .03. Pizza variability was a significant negative predictor of COMPX (Beta = -.26, *t* = -2.1, *p* = .04). Greater pizza variability was associated with lower COMPX scores (see Fig 4). The regression model also found pizza energy content to be a significant positive predictor of COMPX (Beta = .27, *t* = 2.2, *p* = .04). Here, prior experience of consuming higher energy content pizzas was independently associated with higher COMPX scores. Loss aversion was not a significant independent predictor in this model (Beta = -.18, *t* = -1.5, *p* = .15).

**Fig 5:**
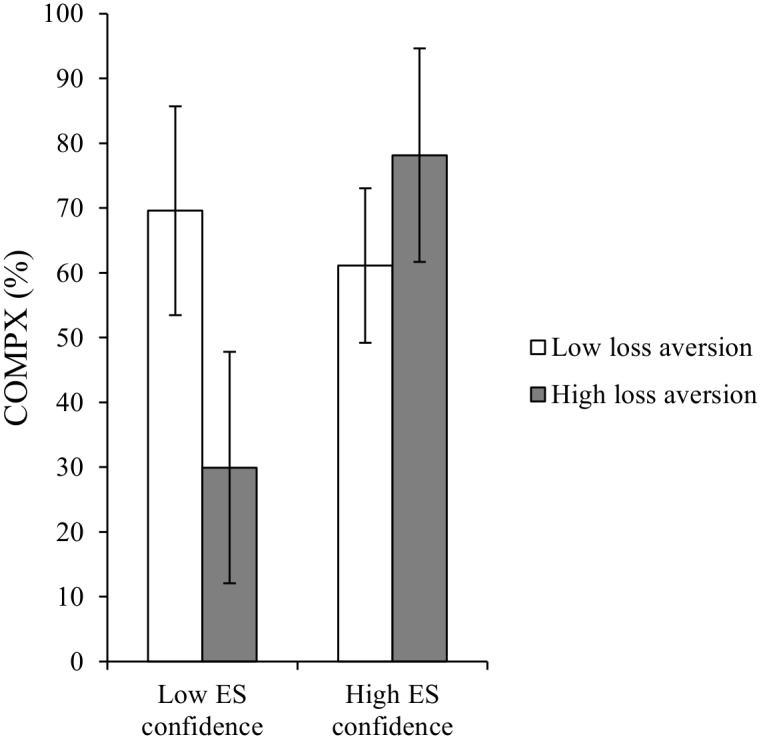
Mean COMPX scores where participants are split by high and low loss aversion and high and low expected-satiation (ES) confidence. Adjusted means were derived from analyses where median splits were taken of the loss aversion and expected-satiation confidence predictors.

The second regression model accounted for 21% of the variance in COMPX, *F*(5, 61) = 3.0, *p* = .018. Pizza variability and pizza energy content remained significant independent predictors of COMPX (Beta = -.26, *t* = -2.1, *p* = .04, and Beta = .27, *t* = 2.2, *p* = .04, respectively). Independently, neither loss aversion nor expected-satiation confidence significantly predicted COMPX (Beta = -.15, *t* = -1.2, *p* = .23; and Beta = .16, *t* = 1.3, *p* = .19, respectively). However, the interaction between expected-satiation confidence and loss aversion was a significant predictor (Beta = .26, *t* = 2.1, *p* = .04). This interaction is presented graphically in Fig 5 (for the purposes of graphical presentation, these adjusted means were derived from analyses based on a median split of expected-satiation confidence and loss-aversion scores). The lowest COMPX scores were found in participants with low levels of expected-satiation confidence and high levels of loss-aversion.

The final model accounted for 25% of the variance in COMPX, *F*(8, 61) = 2.2, *p* = .045. Variability and the interaction between loss aversion and expected-satiation confidence remained significant predictors of COMPX (Beta = -.25, *t* = -2.0, *p* = .049, and Beta = .33, *t* = 2.4, *p* = .019, respectively). No other variables reached statistical significance (all *p* values >.08). This indicates that the association between pizza variability and COMPX cannot be explained by other aspects of prior experience, such as the frequency of pizza consumption, usual pizza portion size, or the number of pizza brands eaten per year.

### Relationship between variability and expected satiation

**Fig 6:**
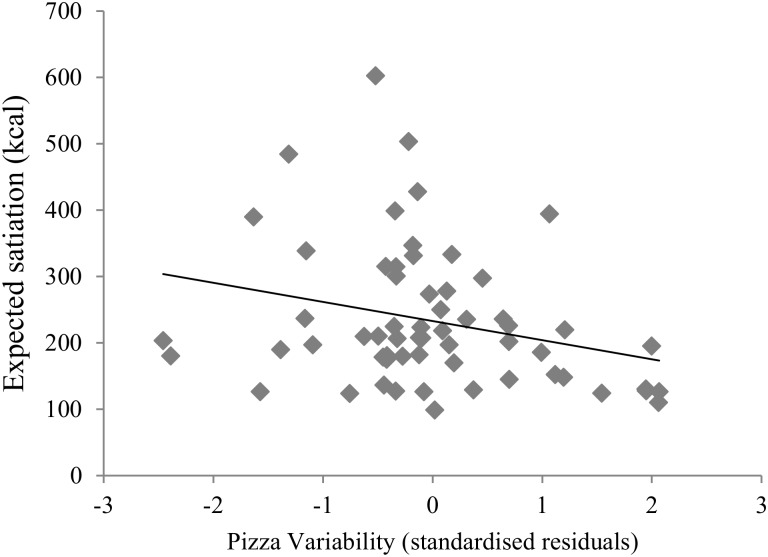
Scatterplot and linear best fit to show the association of pizza variability with expected satiation. Values for pizza variability are standardized residuals adjusted for pizza energy content and loss aversion.

The primary regression model accounted for 12% of the variance in expected satiation, *F*(3, 59) = 2.5, *p* = .07. Pizza variability was a significant negative predictor of expected satiation (Beta = -.28, *t* = -2.2, *p* = .03) (Fig 6). Neither pizza energy content nor loss aversion were significant predictors of expected satiation (Beta = -.09, *t* = -0.72, *p* = .47, and Beta = -.16, *t* = -1.2, *p* = .22, respectively).

The second regression model accounted for 20% of the variance in expected satiation, *F*(5, 59) = 2.6, *p* = .03. Pizza variability remained a significant predictor of expected satiation (Beta = -.30, *t* = -2.4, *p* = .019). None of the other variables (*i*.*e*., pizza energy content, loss aversion, expected-satiation confidence) were significantly associated with expected satiation (all *p* values >.104). The interaction between expected satiation confidence and loss aversion was also non-significant (Beta = -.14, *t* = -1.1, *p* = .28).

The final model accounted for 26% of the variance in expected satiation, *F*(8, 59) = 2.3, *p* = .038. Variability remained the only significant predictor of expected satiation (Beta = -.30, *t* = -2.4, *p* = .019; other variables *p* values >.09). This indicates that the association between pizza variability and expected satiation was not explained by other behaviours such as the frequency of pizza consumption, usual pizza portion size, or the number of pizza brands eaten.

## Discussion

The current study examined the effects of dietary variability in a commonly consumed food (pepperoni pizza) on the control of food intake. To our knowledge, this is the first time that variability in a specific food form has been quantified and considered as a risk factor for overeating in humans. As predicted, higher pizza variability was associated with reduced compensation for calories in pepperoni pizza at a subsequent test meal (*i*.*e*., overeating). Furthermore, variability also influenced expectations about the satiating properties of pepperoni pizza, whereby higher variability was associated with lower expected satiation. Importantly, the associations between pizza variability and the key dependent variables were highly specific and could not be explained by more general aspects of pizza consumption, such as frequency and usual pizza portion size.

This work chimes with the conclusions of Swithers and Davidson. In their studies, the predictive association between orosensory cues and caloric postingestive consequences has been manipulated systematically in non-human animals. They interpret their results as showing that the decoupling of orosensory cues (*e*.*g*., sweet tastes, viscosity, fat) from caloric consequences is responsible for increased food intake, bodyweight, and adiposity [11,12,13,14,15,16,17]. It is possible that the effect of dietary variability in humans reflects such a phenomenon, whereby our ability to use the visual and orosensory properties of a specific food to anticipate its energy content is degraded. In this way, cues in pizza (*e*.*g*., sight, smell, taste) might no longer serve as reliable predictors of pizza energy content. Swithers and Davidson further propose that exposure to non-predictive flavour-nutrient associations results in blunted physiological responses to food (decreased thermic responses and GLP-1 release) which thus account for the disrupted energy intake control [14,16]. However, these previous studies are designed so that basic taste characteristics (*e*.*g*., sweetness) lose their predictive power and this is associated with poor compensation for *novel* sweet foods [14]. In our study, we examined the effects of variability (in energy content) within a specific food that was already familiar to our participants. This is an important distinction and further studies are needed to determine the extent to which our findings might be explained by a mismatch between sensory characteristics and postingestive effects.

Expectations about satiation and satiety are strongly influenced by the orosensory characteristics of a food [46,47]. Furthermore, expected satiation is an excellent predictor of portion-size selection and food intake [24,26,27]. Therefore the relationship we have observed between variability and expected satiation is highly likely to influence food intake. When exposed to a food that has a highly variable energy content, humans may lack the capacity to learn and form expectations about individual brands and varieties. Instead, an expectation might emerge based on an aggregation of experiences with that particular food [10]. In addition, variability is likely to lead to a lack of familiarity with each individual brand and variety of a specific food. This is important because, in previous studies, we have shown that expected satiation increases as a food becomes more familiar [28,29]. In this way, variability might limit the opportunity for this “expected-satiation drift” to occur leading to lower expected satiation for highly variable foods.

An opportunity also exists to explore ways in which our findings fit with a broader literature on decision making and loss aversion. We suggest that dietary variability may generate uncertainty about the postingestive effects of highly-variable foods, and we have quantified this uncertainty using a measure of expected-satiation confidence [28]. On the basis of predictions derived from Prospect Theory [30,31,32], this uncertainty might bias decisions about food intake, especially in individuals who are highly loss-averse. In our study we used a standard monetary gambling task [35] to quantify individual differences in loss aversion. Participants with low levels of expected-satiation confidence and high levels of loss aversion had the lowest COMPX scores. In other words, when loss averse participants were uncertain about the preload’s satiating capacity they tended to consume a larger test meal. This tendency may reflect a precautionary strategy (“playing it safe”) aimed at avoiding the potential negative effects of feeling hungry between meals. To our knowledge, this is the first demonstration that individual differences in loss aversion influence decisions about food intake and therefore have the potential to impact upon health. It is also consistent with findings that the related construct of risk aversion is correlated with obesity [48,49]. Importantly, the effect of loss aversion on decisions around food intake is likely to vary from person to person and across situations. For example, under other circumstances, loss aversion may reflect concerns about the immediate and longer-term negative consequences of overconsumption, such as physiological discomfort and poor health, respectively. A challenge for the future will be to establish exactly how loss aversion manifests itself in decisions around food intake.

We also found greater compensation for calories in the pizza preload in participants who reported consuming higher energy-content pizzas in the past relative to participants who had consumed lower energy-content pizzas. Again, this confirms that the size of the *ad libitum* test meal was governed by prior experience with pizza. It is widely recognised that basic sensory characteristics of a preload can impact subsequent energy compensation [50]. For example, a clear difference is observed between liquids, semi-solids, solids, and composite meals [51]. However, to our knowledge, this is the first study to demonstrate sensitivity based on prior exposure to particular brands of a single food. This finding is noteworthy because it exposes a degree of sophistication that has not been observed previously. Dietary compensation studies show that young children exhibit acute sensitivity to covert manipulations to the energy content of a preload [43,52]; however, this responsiveness decreases with age [39]. This decline may reflect an increasing role for prior experience and learning in the short-term controls of food intake [9]. Our work confirms and extends this hypothesis by suggesting that compensation is governed by prior experience with specific examples of a particular food.

The current study reports the results of cross-sectional associations. In future, it will be important to demonstrate causality, perhaps by systematically manipulating variability within a novel food. It will also be important to consider other food forms and to determine, for example, whether these findings extend to consumption of low-energy sweeteners. The effects of dietary variability that we have observed, while statistically significant, are modest. Nevertheless, the influence of dietary variability repeated over many meals and people could have a substantial population level effect. It would also be informative for future research to examine psychological variables, such as mood and eating behaviour traits, which might moderate the association between dietary variability and food-intake control or predict greater variability per se. For example, our results indicated a positive association between dietary restraint and pizza variability which could be reflective of periods of restriction punctuated by episodes of disinhibited overeating as is characteristic of chronic dieters [53]. Finally, it is noteworthy that poorer countries in the developing world are characterized by a lack of dietary diversity [54]. An opportunity therefore exists to determine whether individuals from these populations show more accurate dietary compensation.

In conclusion, we found that a high level of variability in pizza form was associated with compromised controls of food intake. Variability predicted both reduced compensation for calories in pizza as well as lower expected satiation. In many developed countries variability may be promoted by products that contain low-energy sweeteners and fat substitutes. Our findings suggest that this has the potential to compromise dietary learning leading to overconsumption.
